# The Extract of *D. dasycarpus* Ameliorates Oxazolone-Induced Skin Damage in Mice by Anti-Inflammatory and Antioxidant Mechanisms

**DOI:** 10.3390/antiox7060077

**Published:** 2018-06-15

**Authors:** Tsong-Min Chang, Ting-Ya Yang, Yu-Lin Niu, Huey-Chun Huang

**Affiliations:** 1 Department of Applied Cosmetology and Master Program of Cosmetic Sciences, Hungkuang University, Taichung 43302, Taiwan; ctm@sunrise.hk.edu.tw; 2 Department of Medical Laboratory Science and Biotechnology, College of Medicine, China Medical University, No. 91 Hsueh-Shih Road, Taichung 40402, Taiwan; tyyang@mail.cmu.edu.tw; 3 Niuer International Skincare Science Research Institute, 9F, No. 266, Ruiguang Rd., Neihu Dist., Taipei 11491, Taiwan; niuer80@gmail.com

**Keywords:** *D. dasycarpus*, ROS, interleukin-1β, interleukin-6, tumor necrosis factor-α, NF-κB, inflammasome

## Abstract

*Dictamni dasycarpus* is a type of Chinese medicine made from the root bark of *D. dasycarpus*. It has been reported to show a wide spectrum of biological and pharmacological effects, for example, it has been used widely for the treatment of rheumatism, nettle rash, itching, jaundice, chronic hepatitis and skin diseases. In the current study, *D. dasycarpus* extract was investigated for its antioxidant and anti-inflammatory effects, as well as its capability to alleviate oxazolone-induced skin damage in mice. The possible anti-inflammatory mechanism of *D. dasycarpus* extract against oxidative challenge was elucidated by measuring the levels of reactive oxygen species (ROS) production, interleukin-6, Tumor necrosis factor-α, NLRP3 (NACHT, LRR and PYD domains-containing protein 3 (NALP3)) inflammasome and interleukin-1β in HaCaT cells. *D. dasycarpus* extract did not affect cell viability in basal conditions. The extract significantly reduced oxazolone-induced epidermal swelling compared to untreated animal in the hairless albino mice (ICR mice) model. At the molecular level, Western blot assays indicated that the *D. dasycarpus* extract attenuated oxazolone-induced activation of apoptosis-associated speck-like protein containing CARD (ASC), procaspase-1, NF-κB and mitogen-activated protein kinase (MAPKs) such as c-Jun N-terminal protein kinase (JNK) and p38. This study demonstrates that *D. dasycarpus* extract could protect skin cells against oxidative and inflammatory insult by modulating the intracellular levels of ROS, TNF-α, interleukin-1, interleukin-6, NLR family pyrin domain containing 3 (NLRP3) inflammasome generation, antioxidant enzyme activity and cell signaling pathways. *D. dasycarpus* extract also attenuated the expression of NF-κB in HaCaT keratinocytes and thereby effectively downregulated inflammatory responses in the skin. Furthermore, *D. dasycarpus* extract alleviated oxazolone-induced damage in mice. Our results suggest the potential application of *D. dasycarpus* extract in preventing inflammatory processes in dermatitis.

## 1. Introduction

Traditional Chinese medicine (TCM) therapy used in Taiwan has been enrolled in the National Health Insurance Research Database since 2002 [1,2]. Herbal formulas and single herbs, such as Fang-Feng (*Saposhnikovia divaricate*) and Bai-Xian-Pi (*Dictamni dasycarpus*; *D. dasycarpus*), were prescribed for treating dermatitis [3]. *D. dasycarpus* has been found to possess many interesting pharmacological and physiological activities in the treatment of skin diseases in clinic, although it lacks the mechanistic evidence [4,5]. Phytochemicals such as polyphenols or flavonoids are the most abundant naturally occurring anti-inflammation compounds and have anti-oxidative properties [6,7]. In this study, we studied the protective mechanisms of *D. dasycarpus* against inflamed dermatitis.

The hapten oxazolone-induced dermatitis in mice has been developed as the model for experimental atopic dermatitis or contact dermatitis. Repeated oxazolone applications provoke a pruritic dermatosis with epidermal hyperplasia, erythematous and edematous dermatosis [8,9]. The T cells in the skin tissue sensitize and elicit a hypersensitive inflammation reaction [10], whereas skin keratinocytes provide pro-inflammatory cytokines to initiate and maintain the T cell-mediated immune responses in inflammatory lesions [11,12]. To go through various stages is very important for epidermal keratinocytes to induce adaptive immunity, antigen presentation, keratinocyte activation, and the expression of adhesion molecules. In addition, keratinocytes also act as a potent source of cytokines and chemokines, which result in a particular dialogue between keratinocytes and activated immune cells [13]. In addition, the keratinocytes act as innate immune sensors that synergistically induce of additional inflammation [14,15]. Inflammasomes are multiprotein complexes that assemble in the cytosol after exposure to pathogen-associated molecular patterns (PAMPs) or danger-associated molecular patterns (DAMPs) and result in the activation of caspase-1 and subsequent cleavage of pro-inflammatory cytokines interleukin (IL)-1β and IL-18 [16]. The dysregulated inflammasome activity is associated with numerous skin inflammatory syndromes and skin cancer predisposition [17]. Therefore, attenuating inflammasome activation may provide a valuable therapeutic strategy in treating various inflammation-related disorders [18].

The goal of this study was to investigate the effect of *D. dasycarpus* extract on inflammatory mediators and cellular signaling pathways in oxazolone-challenged mice and HaCaT cells. The topical application of *D. dasycarpus* extract inhibited oxazolone-induced expression of inflammatory mediators. The activation of NF-κB signaling pathways was also investigated. Furthermore, we also elucidated the effect of *D. dasycarpus* extract on oxazolone-mediated NLRP3 activation.

## 2. Materials and Methods

### 2.1. Herb Extraction and Chemicals

The herbs were purchased from Sun Ten Pharmaceutical Co., Ltd. (New Taipei City, Taiwan). *Periostracum cicadae* (PC) and *D. dasycarpus* (DD) were extracted with 50% *v*/*v* ethanol as previously reported [19]. All dried extract was weighed and re-constituted in 0.1% dimethyl sulfoxide (DMSO) containing culture medium to 1 mg/mL. All other chemicals and solvents were obtained from Sigma-Aldrich Inc. (St. Louis, MO, USA).

### 2.2. Animals and Treatments

Mice experiments were conducted in accordance with the International Standards on Animal Welfare and in accordance with the ethical standards of the Laboratory Animal Service Center of China Medical University (Affidavit of Approval of Animal Use Protocol No. 2017-090). Repeated topical application of oxazolone over an extended period was used to induce allergic contact dermatitis in hairless male ICR mice (BioLASCO Taiwan Co., Ltd., Taipei, Taiwan). The mice were subdivided into groups based on their latest body weight (five mice in each group) using a stratified randomization method. Animals were sensitized once with 5% (*w*/*v*) 4-ethoxymethylene-2-phenyl-2-oxazolin-5-one (oxazolone) and dissolved 3:1 in acetone and olive oil (150 μL) on the shaved back. After 3 days, the mice were challenged with 1% oxazolone every other day for 10 days. For the experimental group animals, 5 mg/kg of *P. cicadae* or *D. dasycarpus* was applied onto the mice backs at the day interval. On the other hand, the control group animals were treated with ethanol at the same day interval. All mice were sacrificed and sampled on day 24 [20,21].

### 2.3. Specimens and Immunohistochemistry

After the 24 days of treatment, pentobarbital was administered to all mice (200 mg/kg, IP), and 1 cm^2^ skin tissues were then quickly removed. The skin samples were fixed in 4% buffered neutral formalin solution for 24 h at room temperature, and paraffin was embedded. Serial sections were floated in warm water containing 2% gelatin to prevent peeling off of the sample. The sections were deparaffinized, rehydrated, and stained with hematoxylin and eosin (H&E) for immunohistochemistry. Deparaffinization, re-hydration and, quenching of endogenous peroxidase of the 5 mm paraffin cross-sections were evaluated by 30 min incubation in 0.3% H_2_O_2_ and incubation with 0.5 mg/mL of anti-cyclooxygenase (COX)-2, anti-CD3, anti-CD45 antibodies or control IgG overnight, followed by incubation with biotinylated secondary Abs and developed with the Vectastain Elite ABC kit (Vector) with 3,3-diaminobenzidine (DAB) as a substrate. Examination was done by light microscopy at 200× magnifications.

HaCaT cells were fixed with 4% paraformaldehyde in 250 mM Hepes, pH 7.4, freshly diluted from 16% stocks stored at −20 °C. After standing at room temperature for 5 min, the cells were washed with phosphate-buffered saline (PBS) and treated with blocking solution (PBST, 1% fetal bovine serum, FBS) at 4 °C for 1 h. Cells were incubated with an NLRP3 Ab in blocking solution at 4 °C overnight in a wet chamber. After washing in PBST, the cells were incubated with the appropriate secondary antibody mixtures in blocking solution for at least 1 h at room temperature. The cells were washed three times in PBS and mounted in gold antifade reagent with DAPI (Molecular Probes, Eugene, OR, USA). Confocal analysis was performed using a Leica TCS SP2 confocal microscope: 10 horizontal scans using a 63× (1.3 NA) oil immersion objective were recorded for each image with the imaging software (exported as a TIFF file).

### 2.4. Cell Culture

HaCaT cells were maintained in Dulbecco’s Modified Eagle Medium (DMEM, HyClone; GE Healthcare Life Sciences, Marlborough, MA, USA) supplemented with 10% fetal bovine serum and 1% antibiotics at standard cell culture conditions (37 °C, 5% CO_2_ in a humidified incubator).

### 2.5. Cytokines Measurement

Supernatant obtained from the HaCaT cell culture in the various treatment groups were analysed for interleukin IL-1β, IL-6, IL-8, tumor necrosis factor (TNF)-α with enzyme-linked immunosorbent assay (ELISA) kit (eBioscience, San Diego, CA, USA), according to the manufacturer’s instructions.

### 2.6. Reactive Oxygen Species (ROS) Measurement

HaCaT cells were seeded in 96-well plates at a concentration of 1 × 10^5^ cells/mL. Cells were treated with oxazolone (50 μM), subsequently washed twice with PBS and incubated with 20 μg/mL of *P. cicadae* or *D. Dasycarpus* for 24 h. After being treated with 10 μM 2,7-dichlorofluorescein diacetate (DCFH-DA; Sigma-Aldrich) in PBS for 30 min, the media was discarded, and the cells were washed twice with PBS. The ROS fluorescence were monitored by fluorescence microscopy (Olympus IX71). The mitochondrial ROS (mito ROS) were detected by the Mito SOX^TM^ Red Mitochondrial Superoxide Indicator (Invitrogen, Carlsbad, CA, USA) and monitored by fluorescence microscopy.

### 2.7. Western Blot Analysis

Cells were lysed in PBS containing 1% nonidet P-40, 0.5% sodium deoxycholate, 0.1% sodium dodecyl sulfate (SDS), 5 μg/mL aprotinin, 100 μg/mL phenylmethylsulfonyl fluoride, 1 μg/mL pepstatin A, and 1 mM ethylenediaminetetraacetic acid (EDTA) at 4 °C for 20 min. Total lysates were quantified using a microBCA kit (Thermo Fisher Scientific, Waltham, MA, USA). Proteins (10 μg) were resolved by SDS-polyacrylamide gel electrophoresis and electrophoretically transferred to a PVDF (poly(vinylidene fluoride)) membrane. The membrane was blocked in 5% fat-free milk in PBST (PBS with 0.05% Tween-20), followed by incubation overnight with the following primary antibodies diluted in PBST: JNK Ab, p-JNK Ab, p38 Ab, p-P38 Ab, ASC Ab, procaspase-1 Ab (diluted to 1:1000, all from Santa Cruz Biotech (Dallas, TX, USA)), p65 Ab (Genetex, Taiwan) and NLRP3 Ab (Adipogen, San Diego, CA, USA). The primary antibodies were removed, and the membrane was washed extensively in PBST. Subsequent incubation with horseradish peroxidase-conjugated goat anti-rabbit antibodies (1:20,000, Santa Cruz Biotech) was performed at room temperature for 2 h. The membrane was washed extensively in PBST to remove any excess secondary antibodies, and the blot was visualized with enhanced chemiluminescence reagent (GE Healthcare, South Jakarta, Indonesia).

### 2.8. Statistical Analysis

Statistical analyses were carried out using Student’s *t*-test (sigma plot 10.0. Systat Software, Inc., San Jose, CA, USA) * *p* < 0.05 was considered significant, ** *p* < 0.01, *** *p* < 0.001.

## 3. Results

**Figure 1:**
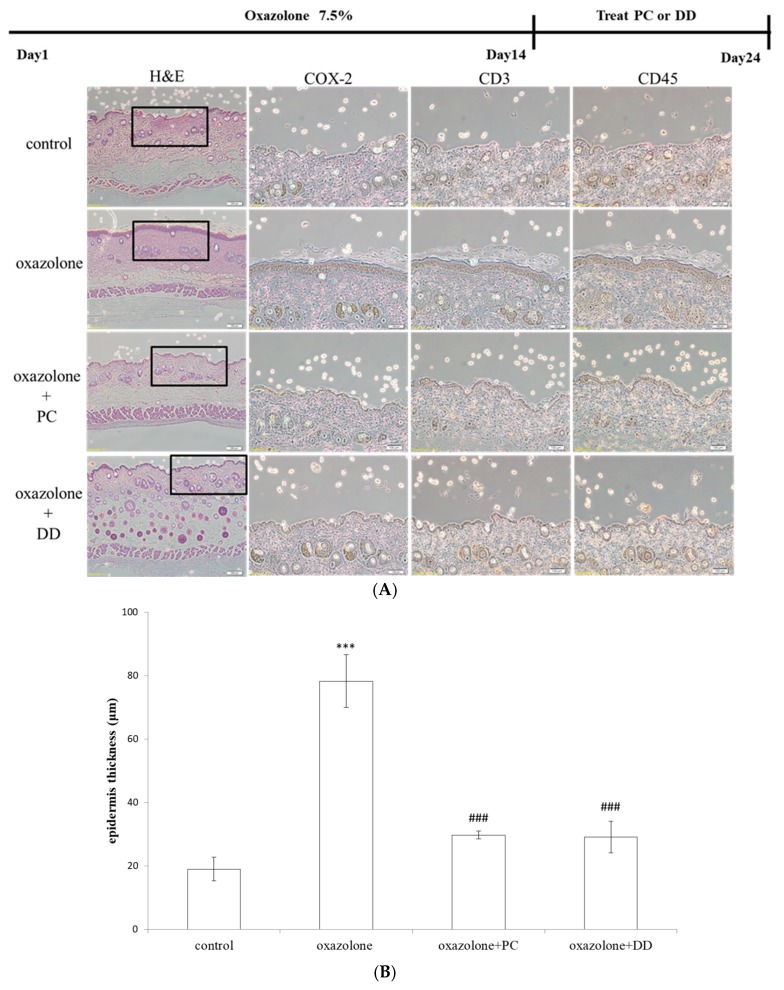
The effect of *P. cicadae* (PC) and *D. dasycarpus* (DD) on oxazolone-induced skin edema. (**A**) Representative scheme showing the treatment of the dermatitis mouse. Histopathological images of *P. cicadae* or *D. dasycarpus* of oxazolone-induced mouse dermatitis. Five-percent oxazolone was applied onto mice back every 2 days, for 10 days. The control group was treated with ethanol instead of oxazolone. Mouse skin was excised after the last application, and a representative section of H&E staining from five mice is shown. Topical treatment 5 mg/kg of *P. cicadae* or *D. dasycarpus* inhibited oxazolone-induced extensive structural damage in both the epidermis and dermis (magnification, 40×). Infiltration of CD3^+^ or CD45^+^ lymphocytes into the dermis and the development of pro-inflammatory component COX-2 were remarkably alleviated in the *P. cicadae* and *D. dasycarpus* treated group. (**B**) Epidermal thickness (μm) was measured histopathologically by H&E staining. Data represents of mean ± SD of three different animals; *** *p* < 0.001, significant when oxazolone-treated group was compared with control; ### *p* < 0.001, significant when PC/CD^+^ oxazolone-treated group was compared to oxazolone only group; COX: cyclooxygenase; CD: cluster of differentiation.

Mouse dorsal skin was sensitized with 5% oxazolone on day 1 and challenged with 1% oxazolone for an additional week at 2-day intervals to develop the acute allergic contact dermatitis. Topical exposure to oxazolone induced a significant skin swelling response and inflammation erythema. The recruitment of inflammatory cells (CD3^+^, CD45^+^ cells) was significantly increased in oxazolone-exposed skin sites. The expression of COX-2, an important mediator of acute phase reactions, was also significantly increased compared with control mice after oxazolone treatment. Histologic evaluation showed keratosis in the stratum corneum and an erosive change in epidermis, mild acanthosis and remarkable loss of hair follicles (epilation), loss of blood vessels and remarkable loss hair of follicles and hypertrophic fat cells, as well as remarkable muscle atrophy compared with the intact structure of the whole skin with no treatment (Figure 1A). Prominent epidermal hyperplasia with abnormal keratinization and hyperkeratosis was quantitated in Figure 1B. The results showed that the oxazolone-treated group had a significantly higher level of skin thickness. The *D. dasycarpus*-treated group showed remarkably decreased oxazolone-induced swelling and erythematic intensity. Similarly, the *P. cicadae* treated group completely prevented UVB-induced damages in the epidermal. In addition, the dermal-epidermal junction returned to near-normal levels compared to that of the control group as noted by the well-marked appearance of dermal papillae and epidermal rete ridges. Moreover, the upper portion of the dermis showed an ordered arrangement of hair follicles. These results indicated that topical administrated *D. dasycarpus* and *P. cicadae* alleviate oxazolone-induced dermatitis at the relative low dose of 5 mg/kg.

**Figure 2:**
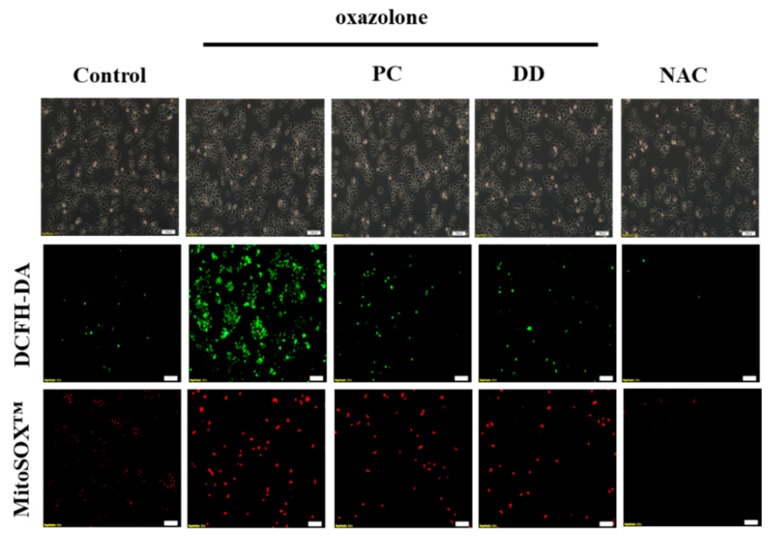
The effect of *P. cicadae* and *D. dasycarpus* on the oxidative levels of HaCaT cells exposed to oxazolone. HaCaT cells were sensitized with oxazolone and treated with *P. cicadae* or *D. dasycarpus*. The generation of cellular ROS were measured by the 2′,7′-dichlorofluorescin diacetate (DCFH-DA), whereas mitochondrial superoxide generation was analyzed by using MitoSOX™ Red as a probe. *N*-acetylcysteine (NAC) was used as an antioxidant control. Representative micrographs (40×) demonstrating. *P. cicadae* and *D. dasycarpus* effectively prevented oxazolone-produced cellular and mitochondrial superoxide in HaCaT cells.

The cultured HaCaT cells were treated with *D. dasycarpus* and *P. cicadae* followed by oxazolone exposure to evaluate the possible mechanism of action of these two Chinese herbs in intracellular stress (Figure 2). The cellular oxygen levels were dramatically increased in the oxazolone-treated cells, whereas the ROS levels were significantly reduced in cells treated with *D. dasycarpus* and *P. cicadae.* In addition, ROS levels in cytoplasm were higher compared to the ROS in mitochondria after treatment with the two herb medicines. These results demonstrated that *D. dasycarpus* and *P. cicadae* can effectively scavenge ROS induced by oxazolone in HaCaT cells.

**Figure 3:**
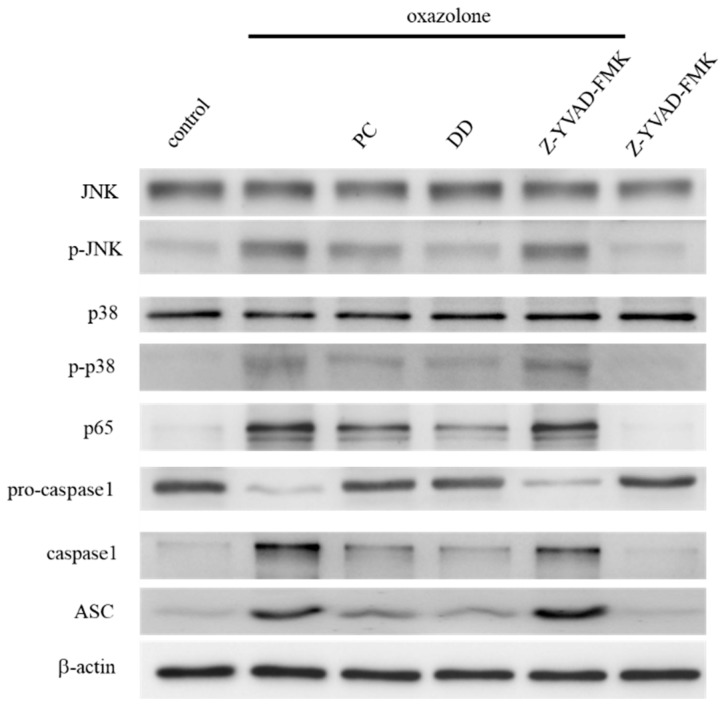
Effects of *P. cicadae* and *D. dasycarpus* on oxazolone-irritated inflammation in HaCaT cells. The HaCaT cells stimulated by oxazolone were cultured with either *P. cicadae* or *D. dasycarpus.* The relative activation of JNK and p38 was determined by Western blotting, and the levels of JNK and p38 were used as an indication of equal loading. Caspase-1, procaspase-1, ASC and p65 expression in HaCaT cells lysates was measured by immunoblotting, whereas β-actin was used as a loading control.

To elucidate the protective mechanism of *D. dasycarpus* and *P. cicadae* on oxazolone-induced allergic contact dermatitis, HaCaT cells were challenged with oxazolone followed by incubation with *D. dasycarpus* and *P. cicadae*. It was found that *D. dasycarpus* and *P. cicadae* showed marked activity against oxazolone-induced expression of p65, procaspase-1 and ASC at 24 h (Figure 3). The inhibitory effect of *D. dasycarpus* was more potent than the effects of *P. cicadae* on p65 production, even though *P. cicadae* has been reported to be an effective inflammatory inhibitor. We examined the effects of *P. cicadae* and *D. dasycarpus* on the activation of the master regulators of antioxidant response, JNK and p38. The results shown in Figure 3 reveal that the inflammation resulted in phosphorylation of the stress-activated MAP kinases p38 and JNK. Cells treated with *P. cicadae* and *D. dasycarpus* exhibited decreased phosphorylation of JNK and p38 compared to cells treated only with oxazolone. The results showed that both herbal medicines reduced the inflammation response of oxazolone-stimulated HaCaT cells through p38 and JNK signaling, which could subsequently decrease IL-6 production.

**Figure 4:**
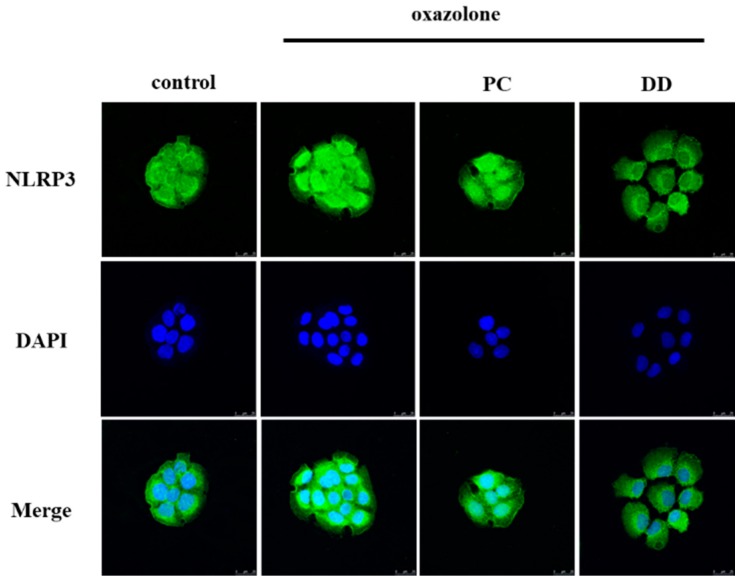
Fluorescent confocal microscopic images showing the localization of inflammasome components NLRP3 (green) in HaCaT cells.

Mitochondria are potential organelles for NLRP3 inflammasome activation because of their vital role in the production of ROS. Western blotting analysis revealed that the 20 kDa form of caspase-1 was present in cell lysates after an overnight treatment with oxazolone. Our results further confirmed that oxazolone could activate the NLRP3 inflammasome and stimulate the maturation of procaspase-1. The *D. dasycarpus* downregulated the capacity of oxazolone, including the inhibition of the expression of procaspase-1 and ASC. The confocal results showed NLRP3 dispersed over the cytoplasm (Figure 4), and the activation state was also confirmed by abundant IL-1β secretion from the same set of cells (Table 1). It was also found that *D. dasycarpus* effectively blocks NLRP3 generation induced by oxazolone.

IL-6 is a marker of activated keratinocytes. As shown in Table 1, oxazolone resulted in dramatic increases in IL-6 protein levels in HaCaT cells. In contrast, treatment with oxazolone plus *P. cicadae* or *D. dasycarpus* significantly reduced IL-6 expression. We further examined the effect of *P. cicadae* and *D. dasycarpus* on IL-8 and TNF-α expression by ELISA. Treatment with *P. cicadae* and *D. dasycarpus* markedly inhibited oxazolone-mediated cytokine expression. The increased levels of IL-1β following oxazolone stress were decreased by pretreatment with the irreversible caspase-1 inhibitor Z-YVAD-FMK (Z-Tyr-Val-Ala-Asp fluoromethylketone). Treatment with *D. dasycarpus* prevented IL-1β expression and *D. dasycarpus* exerted a stronger inhibitory effect than *P. cicadae* (Table 2). Hence, our results demonstrate that the NLRP3 inflammasome is associated with the production of IL-1β after oxazolone stimulation, and *D. dasycarpus* plays a role in a decrease in IL-1β secretion in HaCaT cells treated with oxazolone.

## 4. Discussion

In this study, we investigated the anti-inflammatory activity of *D. dasycarpus*, including the inhibition of the generation of cytokines and the inflammasome, as well as the downregulation of the oxazolone-induced p38 and NF-κB. *D. dasycarpus* displayed in vivo activity at a concentration of 5 mg/kg against oxazolone-induced dermatitis. These results suggest that the *D. dasycarpus* could cease inflammation, which could be achieved via the depletion of ROS and its downstream signaling pathways.

Several compounds have been identified in *D. dasycarpus* extract by pressurized liquid extraction followed by high-performance liquid chromatography [22]. Among these compounds, limonoid and obacunone are the top two abundant triterpenoids, which are well known to possess anti-inflammation beneficial properties [23,24]. In addition, a wide variety of phenylalkylketone compounds derived from herbal products activate potent antioxidant activities, which may also contribute to the dermaprotective effects of *D. dasycarpus* extract on the skin [25].

Oxazolone has been used to induce colitis and contact dermatitis in mice [26]. Our results showed that oxazolone provoked various chemical mediators such as TNF-α, IL-1β, and prostaglandin E2 in the mice skin. In HaCaT cells, oxazolone-induced irritant effects triggered ROS generation and mitochondrial oxidative stress. Recently, oxidative stress has been proposed to play an important role in the activation of the inflammasome [27,28]. Accordingly, ROS are required for NLRP3 inflammasome activation [29,30]. Activation of NLRP3 by extracellular inflammatory insults results in the transcriptional regulation and post-translational modifications of license receptor activation. The indirect immunofluorescence staining of the NLRP3 protein shows a significant overlap of NLRP3 with the endoplasmic reticulum (ER) under non-stimulatory conditions. *D. dasycarpus* could downregulate NLRP3 activation through scavenging ROS, suppressing the expression of ASC, and localizing resting NLRP3 to endoplasmic reticulum structures (Figure 4). Therefore, *D. dasycarpus* limited the assembly of NLRP3 inflammasome, thus preventing procaspase-1 progression, caspase-1 activation and the maturation IL-1β.

**Figure 5:**
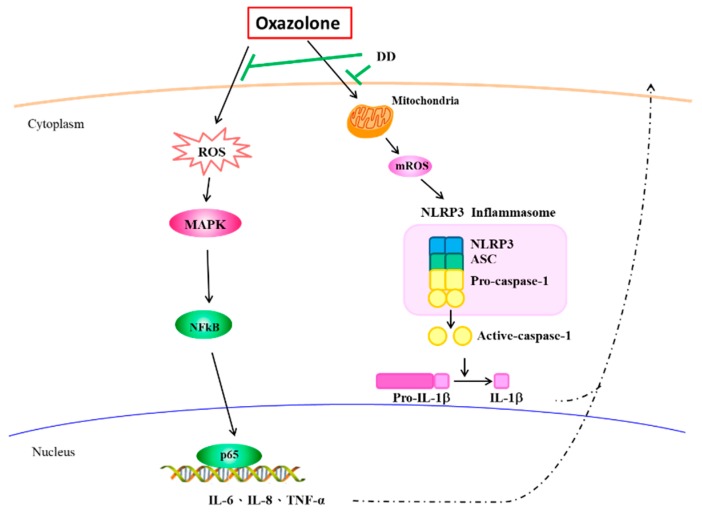
Proposed mechanism by which *D. dasycarpus* abolishes oxazolone-stimulated cytokines production, as well as diminishes NLRP3 inflammasome activation in HaCaT cells.

The inhibition of pro-inflammatory cytokine expression has been reported to improve dermatitis in terms of protection from extended adaptive immunity. We confirmed that oxazolone exhibited dermatitis symptoms, such as erythema and desquamation in the dorsal skin, and has successfully prevented this cutaneous damage in mice by topical administration of *P. cicadae*, as well as *D. dasycarpus. P. cicadae* and *D. dasycarpus* also diminished oxazolone-induced inflammation by decreasing the levels of pro-inflammatory cytokines including IL-6, IL-8, and TNF-α and exhibited signaling regulatory effects in HaCaT cells. *D. dasycarpus* treatment antagonized NF-κB-dependent inflammatory mediators such as IL-1β, IL-6, IL-8, and COX-2 at a protein level since the NF-κB responsive elements have been defined [31,32,33]. Furthermore, our results also showed that *D. dasycarpus* reduced inflammation activity via down-regulation of the p38 and JNK pathways, which could subsequently decrease IL-6 production [34]. *D. dasycarpus* displayed better anti-inflammatory bioactivity than the same concentration of *P. cicadae* upon oxazolone-stimulated in in vivo and in vitro models [19]. Hence, we propose that *D. dasycarpus* could be an antioxidant for therapy against skin exposure to oxazolone (Figure 5).

## 5. Conclusions

We demonstrated both in vivo and in vitro that *D. dasycarpus* protection may involve silencing NLRP3 inflammasome activities and alleviating inflammatory responses. The underlying mechanism was associated with the NF-κB and p38 signaling pathways.

## Figures and Tables

**Table 1:** Cytokine levels in oxazolone-treated HaCaT cells and PC/DD + oxazolone-treated cells.

	Oxazolone			
	Control		PC	DD
TNF-α pg/mL	20.64 ± 0.94	96.51 ± 9.74	87.97 ± 2.35	71.85 ± 4.95 ^a^
IL-6 pg/mL	13.20 ± 2.87	27.40 ± 1.19	14.20 ± 2.43 ^b^	12.30 ± 2.17 ^b^
IL-8 pg/mL	20.64 ± 0.57	198.51 ± 14.24	87.97 ± 3.75 ^b^	51.85 ± 5.21 ^b^

Compared to oxazolone-only group, ^a^ *p* < 0.01; ^b^ *p* < 0.001; TNF: Tumor Necrosis Factor; IL-6: Interleukin 6.

**Table 2:** ELISA analysis showing oxazolone-induced increase in IL-1βproduction in the absence and presence of Z-YVAD-FMK in HaCaT cells. *P. cicadae* and *D. dasycarpus* abolished oxazolone–induced increase in IL-1β.

	Oxazolone						
	Control		PC	DD	Z-YVAD-FMK	Z-YVAD-FMK	
IL-1β pg/mL	25.24 ± 0.57	180.11 ± 14.24	87.67 ± 4.95 ^a^	72.65 ± 7.21 ^a^	57.34 ± 4.54 ^b^	28.84 ± 0.27 ^b^	

Fold of oxazolone, ^a^ *p* < 0.01; ^b^ *p* < 0.001.
